# Medium dependent factors govern the functionality of engineered type III secretion systems

**DOI:** 10.1186/s13036-025-00600-1

**Published:** 2025-12-16

**Authors:** SangKu Yi, Beom Seok Kim, Eunna Choi, Eun-Jin Lee, Juhyun Kim

**Affiliations:** 1 School of Life Sciences, BK21 FOUR KNU Creative BioResearch Group, Kyungpook National University, Daegu, 41566 Republic of Korea; 2 Department of Life Sciences, College of Life Sciences and Biotechnology, Korea University, Seoul, 02841 Republic of Korea

**Keywords:** Type III secretion system, Resource allocation, Translational burden

## Abstract

**Background:**

The type III secretion system (T3SS) is a syringe-like machine that pathogenic bacteria use to inject effector proteins into host cells. Its ability to mediate targeted protein delivery has prompted efforts to adapt it for diverse biotechnological applications. However, the influence of bacterial host culture conditions on the performance of the T3SS-based circuits, which has never been systematically studied, is addressed in this study.

**Results:**

In this study, we developed and characterized an IPTG-inducible, refactored T3SS circuit (iT3SS) in *Salmonella enterica*, in which the *prgH* gene, encoding a protein of the basal body complex, was fused to the coding sequence of GFP in order to monitor the expression of the secretion system. The engineered system was shown to secrete efficiently the effector protein SptP. The dynamics of expression of the PrgH-GFP fusion was assessed in rich LB medium and in glucose minimal medium under various IPTG concentrations. Interestingly, secretion efficiency was maintained across IPTG concentrations in cells grown in glucose minimal medium, but not in cells grown in rich LB medium. In cells grown in LB medium, secretion and invasion efficiencies did not increase proportionally with increasing IPTG concentrations. Both PrgH abundance and SptP secretion efficiency were lower at high IPTG concentration than at low and medium IPTG concentrations. Since RNA-seq analysis of cells grown in LB medium revealed that the transcription of iT3SS genes increased proportionally to inducer level, this indicated that transcription was not the limiting factor for iT3SS expression. This suggested that the limiting factor might be due to a translational and/or post-translational burden of iT3SS component mRNAs. Indeed, uneven (not stoichiometric) translation of the iT3SS components and/or their imperfect folding might impair their assembly and insertion in the membrane. Consequently, one cannot exclude that the iT3SS components not properly assembled or integrated are being degraded, giving the wrong impression of a low translation level. Interestingly, RNA-seq revealed that in LB cultures at high IPTG concentration, stress-response genes were up-regulated whereas ribosomal protein-coding genes were down-regulated. This feature might contribute to limiting iT3SS translation. Several hypotheses are proposed in the discussion to explain how culture conditions could influence the functionality of iT3SS.

**Conclusions:**

Our findings demonstrate that the nature of the growth medium has an impact on the performance of programmable secretion systems that might be due to host’s resource-allocation strategy that would have a negative impact on the translational efficiency of the iT3SS components, compromising their correct assembly and thus their membrane insertion. This insight provides a medium-aware framework for optimizing engineered secretion platforms for synthetic biology applications.

**Supplementary Information:**

The online version contains supplementary material available at 10.1186/s13036-025-00600-1.

## Background

The type III secretion system (T3SS) is a sophisticated syringe-like nanomachine used by gram-negative bacteria to translocate effector proteins directly into eukaryotic host cells [1–4]. This system plays crucial roles in bacterial pathogenesis by facilitating the evasion of host immune defenses and manipulation of intracellular signaling pathways, particularly during early infection stages [5–7]. Thus, the T3SS significantly contributes to bacterial survival, bacterial colonization, and disease progression [8–10]. This system has been extensively studied at both structural and mechanistic levels [11, 12]. For instance, the T3SS of *Salmonella* sp. consists of a needle-like channel that spans the host membrane, and thereby enables the translocation of effector proteins into the host cytosol [13, 14]. These effectors, which possess specific secretion signals, are guided through the system with the help of dedicated chaperones such as SicP, SicA, and InvB. These chaperones stabilize their cognate effector proteins in the bacterial cytoplasm and facilitate their recognition by the secretion apparatus [15–17]. In addition, the translocators SipB, SipC, and SipD assemble at the tip of the secretion needle to form the translocon pore, enabling the delivery of effector proteins across the host cell membrane. Together, these components ensure efficient assembly and functionality of the secretion system.

By harnessing the secretion system, many research groups have repurposed the T3SS for diverse biotechnological applications. Widmaier et al. used the *Salmonella* T3SS to produce proteins that require extracellular localization and avoid cytoplasmic aggregation [18]. They engineered target proteins, including a small human protein (DH domain) and spider silk monomers, by fusing them to an N-terminal secretion tag and coexpressing them with specific chaperones [18]. In therapeutic contexts, bacteria armed with the T3SS have been engineered to deliver apoptosis-inducing or cell cycle-disrupting proteins directly into the cytoplasm of target mammalian cells, enabling rapid and selective killing of cancer cells [19]. Moreover, engineered bacteria equipped with T3SS injectosomes can deliver immunomodulatory cytokines or apoptosis-inducing factors, thereby enhancing therapeutic precision while reducing systemic side effects [19–21]. These applications exploit the inherent protein delivery capabilities of the T3SS and highlight its potential as a programmable protein secretion platform in synthetic biology.

Despite these significant advancements, the use of the T3SS in synthetic biology remains challenging because the assembly of the secretion apparatus is influenced by bacterial gene expression pattern. Bacteria carry a tiered regulatory hierarchy for T3SS expression. This allows the cells to express the secretion system only when necessary [22–24]. For example, in *Salmonella* Typhimurium, the expression of the secretion system is tightly controlled by a hierarchical regulatory cascade initiated by HilD, which activates HilA and the InvF–SicA complex in response to environmental cues such as osmolarity, oxygen level, and host cell contact [25–27]. Although this stringent regulation ensures proper timing during infection, it renders the system highly context-dependent and unsuitable for biotechnological purposes [28]. Therefore, to enable tunable secretion of heterologous proteins, the system needs to be rewired for host-independent regulation. Strategies to engineer native regulation-independent T3SS circuits have been developed to overcome these barriers [24, 29, 30]. A prominent example is the construction of minimal T3SS circuits containing only essential structural genes [31]. Such simplified platforms are uncoupled from bacteria-embedded regulatory systems and enable direct and precise control of T3SS expression, enhancing predictability and secretion efficiency. Despite previous efforts to engineer the T3SS for heterologous protein secretion, the broader biological consequences of altering its expression remain largely unexplored. In particular, how different T3SS expression levels impact cellular resource allocation—and thereby influence overall cellular economics—has not been systematically investigated. Given that bacteria possess limited transcriptional and translational resources, such as RNA polymerase and ribosomes [32–34], understanding how these resources are distributed under varying secretion demands is crucial to optimizing system performance.

In this study, we assessed the relationship between T3SS expression levels, protein secretion efficiency, and cellular physiology in a controlled synthetic context. We constructed an inducible, isopropyl β-D-1-thiogalactopyranoside (IPTG)-responsive circuit to enable the precise modulation of secretion levels. Using this modular approach, we quantitatively measured how different induction intensities influence secretion efficiency and cellular injection capacity. Interestingly, when cells were grown in minimal medium, secretion efficiency was proportional with IPTG concentrations, whereas in nutrient-rich LB medium, secretion and invasion efficiencies declined at high IPTG levels. RNA-seq analysis of LB cultures confirmed that transcription of the iT3SS genes increased proportionally with IPTG concentration, indicating that reduced transcription was not responsible for the lower secretion efficiency. Rather, the limitation may arise from translational or post-translational burden. Uneven (non-stoichiometric) translation of iT3SS components or their improper folding could impair assembly and membrane insertion, and extensive degradation of misassembled components could give the false impression of reduced translation. Altogether, these results demonstrate that medium-specific resources govern iT3SS performance and provide direction for the optimization of protein-delivery systems.

## Materials and methods

### Bacterial strains and culture conditions

The *Escherichia coli* and *Salmonella* strains used in this study are listed in Table S1. Bacterial cells were cultured in lysogeny broth (LB, Cat. #244620, BD, USA) at 37 °C with shaking at 180 rpm, unless stated otherwise. For solid culture, LB agar plates containing 1.5% (w/v) agar were used. When necessary, antibiotics were added to the growth media at the following final concentrations to facilitate transformation and maintain plasmid selection: gentamicin (Gm, Cat. #G1264, Merck, USA) at 150 µg/mL and kanamycin (Km, Cat. #5125 − 2225, Daejung, Korea) at 50 µg/mL. For host cell invasion assays, extracellular bacteria were removed by treatment with gentamicin (Cat. #ML003-03, Welgene, Korea) at 120 µg/mL for 1 h. Chemical inducers were used to regulate plasmid expression where indicated. Isopropyl β-D-1-thiogalactopyranoside (IPTG, Cat. #I6758, Sigma-Aldrich, USA) was used at final concentrations of up to 1 mM, typically prepared as a 100-fold stock solution in water. 3-Methylbenzoate (3 MB, Cat. #T36609, Sigma-Aldrich, USA) was added to cell culture media at a final concentration of 1 mM from a 500 mM stock solution. All inducers and antibiotics were freshly prepared or stored according to the manufacturers’ instructions and added immediately prior to use.

### Cloning procedures and construction of strains

All primers used in this study are listed in Table S2. *S.* Typhimurium 14028s ΔSPI-1 strain was generated by the one-step gene inactivation methods [35]. A Km^R^ cassette for the SPI-1 genes (*hilD*-*invH*) was PCR amplified from plasmid pKD4 using primers Del-SPI-1 hilD-F/Del-invH-R. The resulting PCR product was integrated into the 14028s chromosome to generate ΔSPI-1 (ΔSPI-1::Km^R^) [36].

The inducible T3SS system was constructed by selecting essential genes for the secretion apparatus as described in a previous study [31]. Six DNA fragments constituting the secretion apparatus module, including *prgH* fused to *msfGFP*, were chemically synthesized (Integrated DNA Technologies gBlock, USA). These fragments, designed with overlapping homology regions, were assembled into the plasmid pSEVA234, pre-digested with *Avr*II and *Not*I, using the NEBuilder HiFi DNA Assembly Kit (Cat. #E2621S, New England Biolabs, USA) to generate pSEVA234::iT3SS.

To construct the effector expression system, the *sptP* gene was PCR-amplified from *S.* Typhimurium 14028s chromosomal DNA using the primer pair SptP-F/R. The resulting amplicon, which included an HA tag coding sequence, was digested with *Bam*HI and *Hind*III and subsequently ligated into the pSEVA658 vector, yielding the construct pSEVA658::SptP-HA.

To investigate the secretion of a polymer-hydrolyzing enzyme via the T3SS, the cutinase expression system was engineered to incorporate a T3SS-specific secretion signal. The coding sequences of *cutinase* and its cognate T3SS-specific chaperone, *SicP*, were PCR-amplified using the primer pairs SicP-F/R and TfCut2-F/R, respectively. The ribosome binding site BBa_B0034 was incorporated upstream of the cutinase gene through the use of primers with designed overhangs. The assembled fragments were cloned into the pSEVA658 vector via isothermal assembly (NEBuilder HiFi DNA Assembly Kit, Cat #E2621S, New England Biolabs, USA).

We also constructed the prgH expression system. To this end, the *prgH* gene was PCR-amplified from the pSEVA234::iT3SS plasmid using primers (prgH-F/R) designed to incorporate a standard ribosome binding site (BBa_B0034) upstream of the *prgH* gene. The resulting fragment was cloned into the pSEVA234 vector using *Bam*HI and *Hind*III restriction sites.

All plasmid constructs were verified by colony PCR, Sanger sequencing, or Oxford nanopore whole plasmid sequencing, depending on the target size.

### Measurement of bacterial growth and expression of reporter proteins

Reporter strains expressing green or red fluorescent proteins were pre-cultured overnight in either LB or M9 medium supplemented with the appropriate carbon sources. The cultures were then diluted 1:100 into 24-well microplates (flat-bottom, clear-walled; Cat. #30024, SPL Life Sciences, Korea) containing the same medium with varying IPTG concentrations, as specified. The samples were incubated for 24 h, after which fluorescence was measured at 488/525 nm for GFP and 545/591 nm for RFP using the microplate reader. Cell density was determined by measuring OD600.

### Microscopy and image analysis

Bacterial samples were immobilized by depositing them onto cover slips coated with poly-L-lysine (Cat. #P4707, Sigma-Aldrich, USA) and stored at room temperature to dry. The coverslip was then assembled with a slide glass containing the antifade reagent Prolong (Cat. #P36984, Thermo Fisher Scientific, USA) and sealed with clear nail polish. The sample was visualized using a fluorescence microscope. Microscopy was performed using an Olympus BX53F2 apparatus equipped with an x100 phase contrast objective and an FX900C camera of the same brand. GFP signals were visualized using U-FGFP filters under wide-field illumination. Image acquisition and processing were performed using the software ImageJ and Fiji.

### Western blot analysis of SptP effector levels

Overnight cultures of the WT, ΔSPI-1, or *E. coli* MG1655, each harboring pSEVA658::SptP-HA, with or without pSEVA234::iT3SS, were diluted 1:20 into fresh media containing varying concentrations of IPTG and incubated for 3 h. After induction, 3 MB was added to a final concentration of 1 mM, followed by an additional 1 h of incubation under the same conditions. All cultures were adjusted to an OD600 of 1.0, and cell-free supernatants were collected by filtration through 0.2-µm pore-size membranes. The supernatants were precipitated with 20% trichloroacetic acid (TCA, Cat. #T0699, Sigma-Aldrich, USA) at 4 °C for 10 min and centrifuged at 20,000 ×g for 15 min at 4 °C. Pellets were washed three times with ice-cold acetone, air-dried, and resuspended in 2× Laemmli sample buffer (Cat. #S3401, Sigma-Aldrich, USA). To determine intracellular concentrations of both SptP-HA and DnaK, cells were lysed using B-PER bacterial protein extraction reagent (Cat. #89821, Thermo Fisher Scientific, USA), according to the manufacturer’s instructions. Lysates were mixed 1:1 with 2× Laemmli sample buffer and boiled at 95 °C for 5 min.

Prepared protein samples were separated by SDS–PAGE using NuPAGE 4–15% TGX precast gels (Cat. #456–1084, Bio-Rad, USA) in SDS-containing buffer, and transferred to polyvinylidene difluoride (PVDF) membranes using the Trans-Blot Turbo Transfer Pack and Transfer System (Cat. #1704156 and #1704150, respectively; Bio-Rad, USA). Membranes were blocked in Tris-buffered saline with 0.1% Tween-20 (TBST) containing 5% skim milk for 1 h at room temperature, then incubated overnight at 4 °C with primary antibodies: anti-HA (Cat. #71-5500, Thermo Fisher Scientific, USA) and anti-DnaK (Cat. #PA5-117658, Thermo Fisher Scientific, USA), diluted 1:2000 and 1:4000, respectively, in TBST with 5% skim milk.

Following three 5-minute washes in TBST, membranes were incubated with HRP-conjugated goat anti-rabbit secondary antibody (Cat. #31460, Thermo Fisher Scientific, USA) diluted 1:20,000 in TBST containing 5% skim milk for 1 h at room temperature. Excess secondary antibody was removed by three additional 5-minute washes in TBST. Chemiluminescent signals were detected using the SuperSignal West Pico PLUS Chemiluminescent Substrate (Cat. #34579, Thermo Fisher Scientific, USA) and imaged using the ImageQuant LAS 500 Imaging System (GE Healthcare, USA). Band intensities were quantified using ImageJ software.

Relative secretion levels were calculated by comparing the band intensities to those of a control strain processed in parallel.

### HeLa cell culture and invasion assays

HeLa cells were cultured in DMEM (Cat. #LM001-01, Welgene, Korea) supplemented with 10% heat-inactivated FBS (Cat. #S001-01, Welgene, Korea) at 37 °C in a humidified incubator under 5% CO₂. Subsequently, 5 × 10⁵ cells were seeded into 24-well cell culture plates and incubated for 20 h to form confluent monolayers. Various *S.* Typhimurium 14028s strains were used for infection, including the WT strain (positive control), the ΔSPI-1 (negative control), and four plasmid-bearing WT strains—pSEVA234::iT3SS, pSEVA234::sfGFP, pSEVA234::prgH, and pSECRi::prgH—used as experimental samples for infection at a multiplicity of infection (MOI) of 100.

Overnight bacterial cultures were diluted 1:20 into fresh LB broth containing IPTG (final 1:100), and incubated for 3 h until OD600 ≈ 1.0. These cultures were then diluted in prewarmed DMEM and added to the 24-well plates containing HeLa monolayers. To facilitate contact between bacteria and host cells, the plates were briefly centrifuged at 300 × g for 3 min. The gentamicin protection assay was performed to assess bacterial invasion, with a 30-minute infection at 37 °C under 5% CO₂.

After infection, the wells were washed twice with prewarmed DPBS (Cat. #LB001-01, Welgene, Korea) and incubated for 90 min in DMEM containing 120 µg/mL gentamicin at 37 °C under 5% CO₂ to eliminate extracellular bacteria. The wells were then washed again twice with prewarmed DPBS, followed by the addition of 500 µL of 1% Triton X-100 (Cat. #HC0694, Hanlab, Korea) for 10 min to lyse the HeLa cells. Released bacterial cells were collected, serially diluted in DPBS, spread onto LB agar plates, and incubated at 37 °C for 16 h to determine colony-forming units (CFUs).

### Competitive growth assay

For competitive growth assays, *S.* Typhimurium 14028s strains carrying either pSEVA234::RFP or pSEVA234::iT3SS were used. Each strain was grown overnight in LB or M9 minimal medium supplemented with glucose, then adjusted to an OD600 of 1.0. Equal cell masses of the RFP- and iT3SS-expressing strains were mixed and diluted 1:100 into the same medium used for the overnight culture, supplemented with the appropriate IPTG concentration. The mixtures were inoculated into 24-well microplates for incubation. The mixed cultures were incubated for 24 h at 37 °C with shaking at 230 rpm using a microplate shaker (Jeio Tech, Korea). After incubation, the relative abundance of each strain was determined by flow cytometry (NovoCyte Advanteon, Agilent Technologies, USA) based on RFP and GFP fluorescence signals. Due to the bistable expression of the iT3SS circuit, only a subset of iT3SS-expressing cells showed detectable GFP signals. Therefore, RFP-positive cells (representing the WT strain) were used to define the proportion of the WT population, and the RFP-negative fraction was considered to represent the iT3SS-expressing strain. The ratio of RFP-negative to RFP-positive cells was used as a quantitative measure of competitive fitness.

### Genome-wide transcriptome analysis

Overnight cultures of *S.* Typhimurium 14028s harboring pSEVA234::iT3SS were diluted 1:100 into 3 mL of LB medium, and IPTG was added to final concentrations of 20 µM and 200 µM. The 20 µM condition was chosen as a representative moderate induction level, as it provided detectable T3SS expression and secretion activity while minimizing growth defects that appeared at higher IPTG concentrations. The cultures grew at 37 °C with shaking until they reached the exponential phase (OD600 ≈ 0.4), and 1 mL of each culture was collected for RNA extraction. To preserve RNA integrity, cell pellets were resuspended in ice-cold phenol/ethanol solution (5% water-saturated phenol in ethanol). The cells were then lysed in 2 mg/mL lysozyme prepared in Tris-Cl buffer (pH 7.5) at 37 °C for 10 min. Total RNA was extracted using the RNeasy Mini Kit (Qiagen, Germany) according to the manufacturer’s instructions. RNA quality and quantity were assessed using the TapeStation 4200 system and the Epoch Microplate Spectrophotometer (Agilent Technologies, USA).

Library preparation and RNA sequencing were outsourced to CJ Bioscience (Korea). Libraries were constructed using the Illumina Stranded Total RNA Prep with Ribo-Zero Plus Microbiome kit (Illumina, USA), and sequenced on the Illumina NovaSeq6000 platform using paired-end 150 bp reads. Image analysis was performed using NovaSeq Control Software v1.3.1, and base calling was followed by demultiplexing with bcl2fastq v2.20.0.422 to generate FASTQ files. Quality-filtered reads were aligned to the *S.* Typhimurium 14028s reference genome (NCBI accession: GCA_000022165.1) using Bowtie2. Transcript abundance was calculated in FPKM (fragments per kilobase of exon per million mapped reads).

### Statistical analysis

Statistical analyses were performed using GraphPad Prism (v10.4.2). Depending on the experimental design, unpaired two-tailed Student’s *t*-tests or one-way or two-way analysis of variance (ANOVA) followed by Dunnett’s multiple comparison test were applied. Statistical significance was defined as follows: *P < 0.05, **P < 0.01, ***P < 0.001, ****P < 0.0001, and ns indicates not significant (*P* > 0.05).

## Results

### An inducible iT3SS circuit exhibits functionality defects at high inducer levels in rich LB medium

**Fig. 1:**
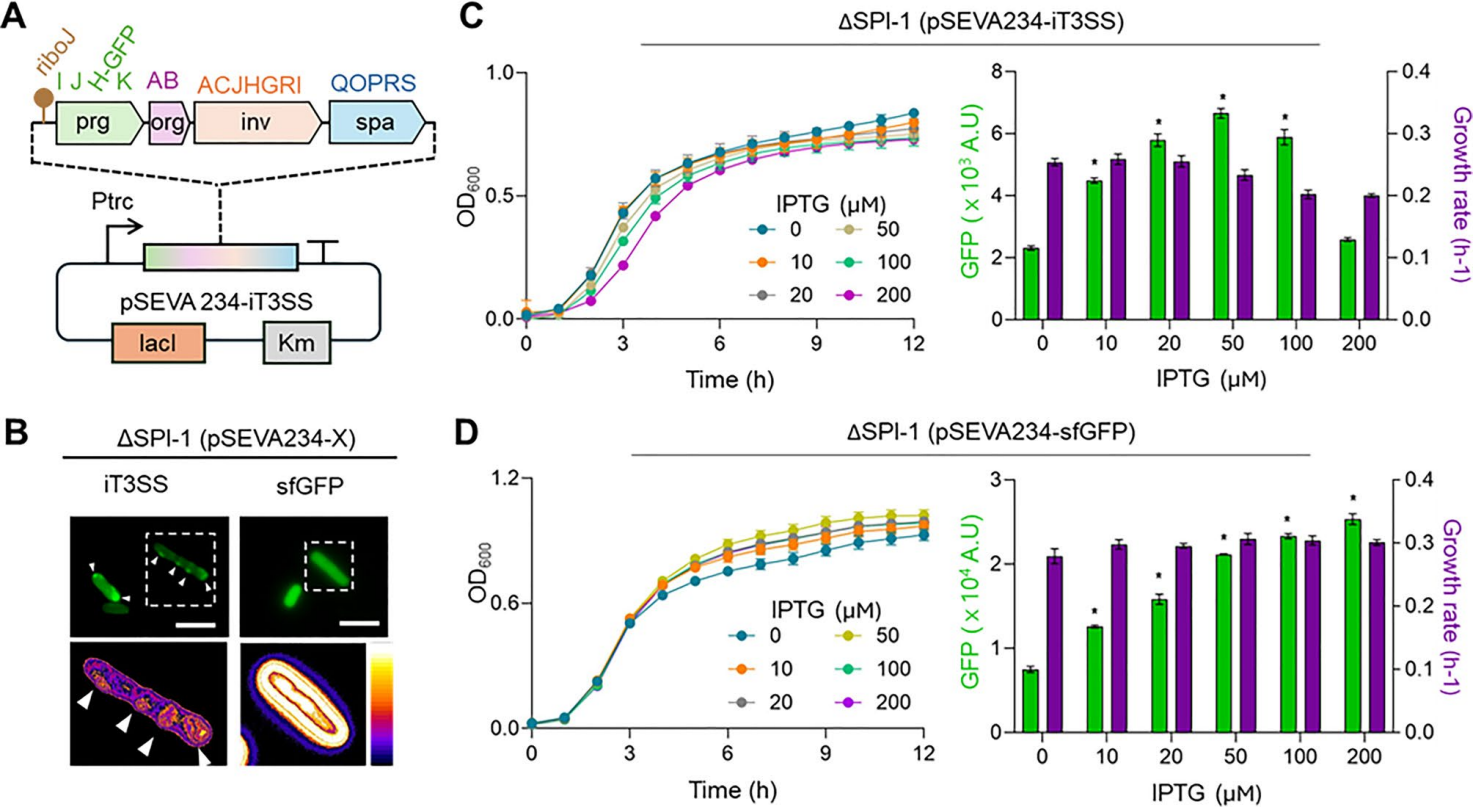
Construction and characterization of the inducible T3SS (iT3SS) system (**A**) Schematic representation of the iT3SS genetic circuit. Based on a previously reported refactored secretin system [31], the essential T3SS genes were assembled into a single operon regulated by an IPTG-inducible promoter. To monitor system expression, the reporter gene *msfGFP* was translationally fused to the inner membrane ring component *prgH*. (**B**) Fluorescence microscopy images of reporter strains expressing either prgH-msfGFP from the iT3SS construct or cytoplasmic sfGFP. Cells were cultured in LB supplemented with 20 µM IPTG for 12 h, and samples were prepared for microscopy. The lower panels show fluorescence intensity maps generated in Fiji, highlighting the spatial distribution of PrgH–GFP signals within the cells. White arrowheads indicate representative peripheral fluorescence foci corresponding to PrgH localization. Scale bars = 5 μm. (**C**, **D**) Monitoring of growth and fluorescence in the ΔSPI-1 strain expressing iT3SS or sfGFP. The ΔSPI-1 strain carrying either pSEVA234::iT3SS (**C**) or pSEVA234::sfGFP (**D**) was cultured in LB supplemented with varying IPTG concentrations. Optical density (OD600) and GFP fluorescence were measured hourly over a 12-hour period. The bar graph indicates GFP fluorescence levels at 12 h (Green bar) and corresponding growth rates (Purple bar), calculated from the growth curves. Error bars represent the mean ± SD (*n* = 3). Statistical significance was determined by two-way ANOVA followed by Dunnett’s multiple-comparison test. Asterisks indicate significance relative to the 0 µM IPTG control. *P < 0.05. Bars without asterisks are not significant (*P* > 0.05)

Building on the previously described simplified T3SS expression system [31], we cloned the essential genes encoding the secretion apparatus under the control of an IPTG-inducible promoter. To achieve this, DNA fragments encompassing the *prgIJHK*, *orgAB*, *invACJHGRI*, and s*paQOPRS* clusters were synthesized and assembled into the pSEVA234 backbone (Fig. 1A). These clusters encode, respectively, the needle complex, basal body, cytoplasmic ATPase, and export gate components that together constitute the core of the T3SS machinery required for apparatus assembly and efficient protein secretion [1]. During DNA assembly, we incorporated a synthetic 5′-UTR element, RiboJ [37] upstream of the T3SS operon (Fig. 1A) to enhance its expression efficiency. Furthermore, *prgH* [38, 39] was fused to the gene encoding the monomeric superfolder Green Fluorescent Protein GFP (msfGFP) to monitor the expression and localization of the secretion system. This construct was designated pSEVA234::iT3SS (hereafter referred to as iT3SS). In parallel, the gene encoding a cytoplasmic GFP was cloned into pSEVA234, yielding pSEVA234::sfGFP, to assess intracellular GFP reporter distribution. The two constructs were introduced into an *S.* Typhimurium ΔSPI-1 strain devoid of the chromosomal genomic island that encodes the T3SS [2], thus eliminating native background secretion. Fluorescent microscopy revealed that PrgH–GFP localized to the periplasmic region, consistent with proper assembly of the basal body [11, 40], while *sfGFP* remained cytoplasmic (Fig. 1B).

**Fig. 2:**
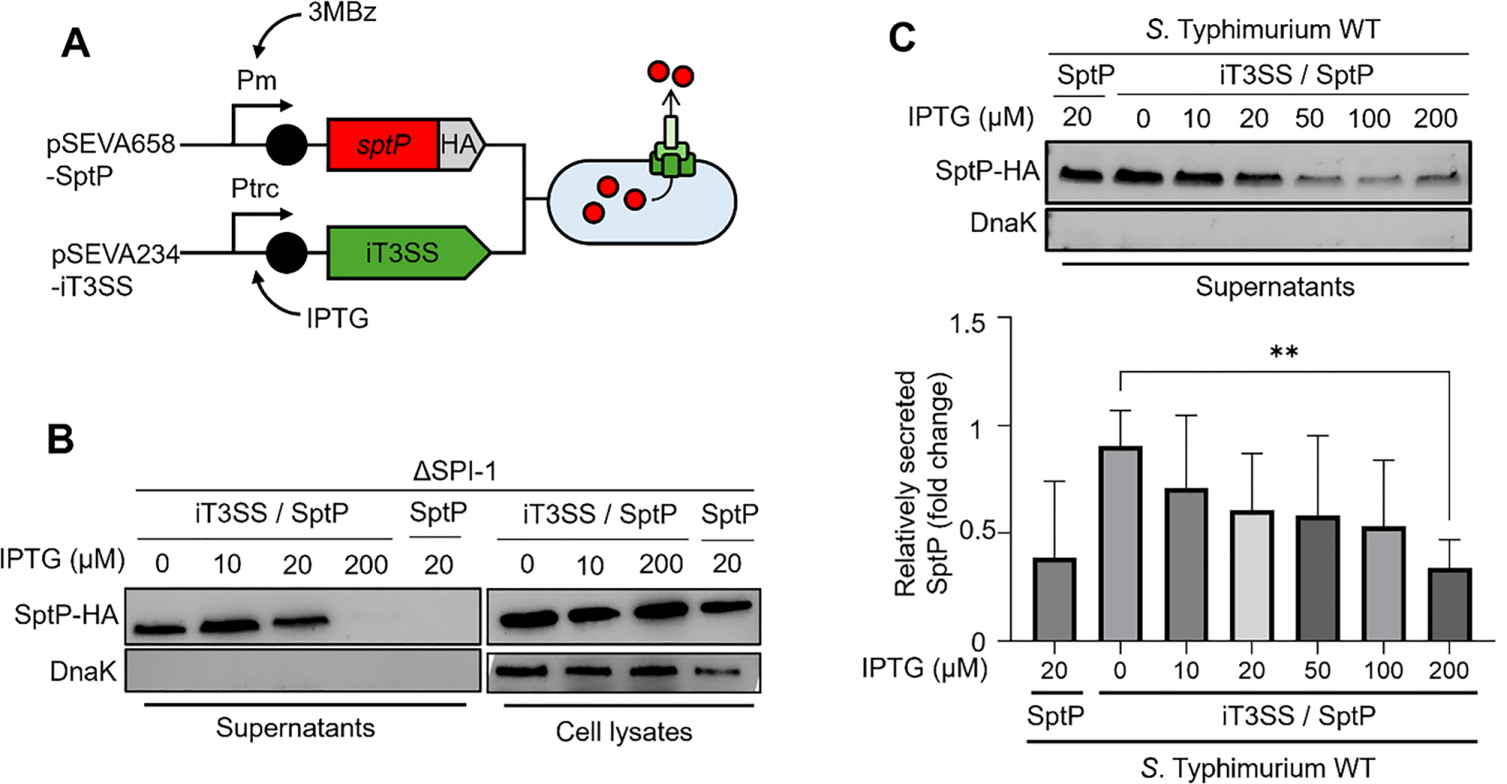
Effect of iT3SS induction on SptP secretion efficiency (**A**) Schematic illustration of the SptP effector secretion system. The SptP coding sequence was translationally fused to a 2× HA tag, and its expression is driven by the 3-methylbenzoate (3 MB)-inducible system. The resulting fusion protein is secreted in response to the expression level of the iT3SS machinery. (**B**) Western blot analysis to detect the SptP effector. The ΔSPI-1 strain harboring both the iT3SS and SptP expression systems was cultured to the exponential phase in LB supplemented with varying concentrations of IPTG to induce iT3SS machinery expression, followed by 3 MB treatment to activate SptP expression. To assess both intracellular and extracellular SptP levels, cell lysates and cell-free supernatants were analyzed. DnaK was used as a loading control and cytoplasmic marker for both fractions. Control strains lacking the iT3SS showed no detectable secretion of SptP-HA. (**C**) Western blot analysis of secreted SptP levels in response to varying iT3SS expressions. *S*. Typhimurium WT strains carrying the iT3SS were grown to the exponential phase in LB with varying concentrations of IPTG (0–200 µM). The bar graph shows densitometric analysis of SptP-HA levels from the western blot, normalized to the highest signal detected in each secretion assay. Values represent mean ± SD (*n* = 3). Statistical significance was determined by an unpaired Student’s *t*-test (**P < 0.01)

**Fig. 3:**
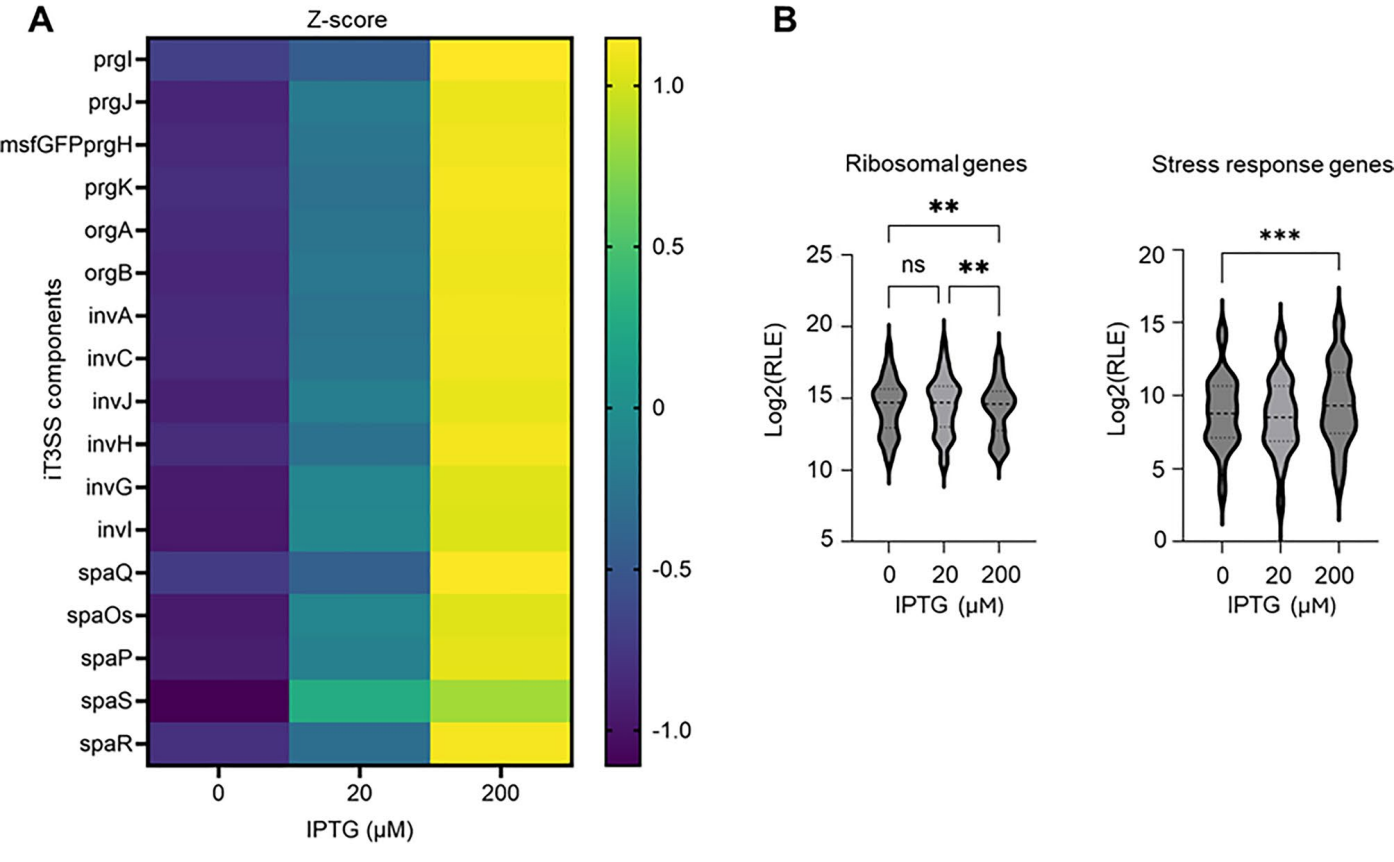
Consequences of iT3SS induction on cellular transcriptome (**A**) Z-score–normalized RNA-seq expression of genes constituting the iT3SS (*org*,* prg*,* inv*, and *spa* clusters, including *prgH-msfGFP*) under 0, 20, and 200 µM IPTG. (**B**) Violin plots showing log_2_-transformed relative log expression (RLE) values of ribosomal protein–encoding genes and stress response genes. *S.* Typhimurium WT strains carrying the iT3SS system were cultured in LB medium supplemented with the indicated IPTG concentrations, and total RNA was extracted for RNA-seq analysis. Statistical significance was determined using one-way ANOVA with Tukey’s multiple comparison test. **P < 0.01, ***P < 0.001, and ns: not significant (*P* > 0.05)

**Fig. 4:**
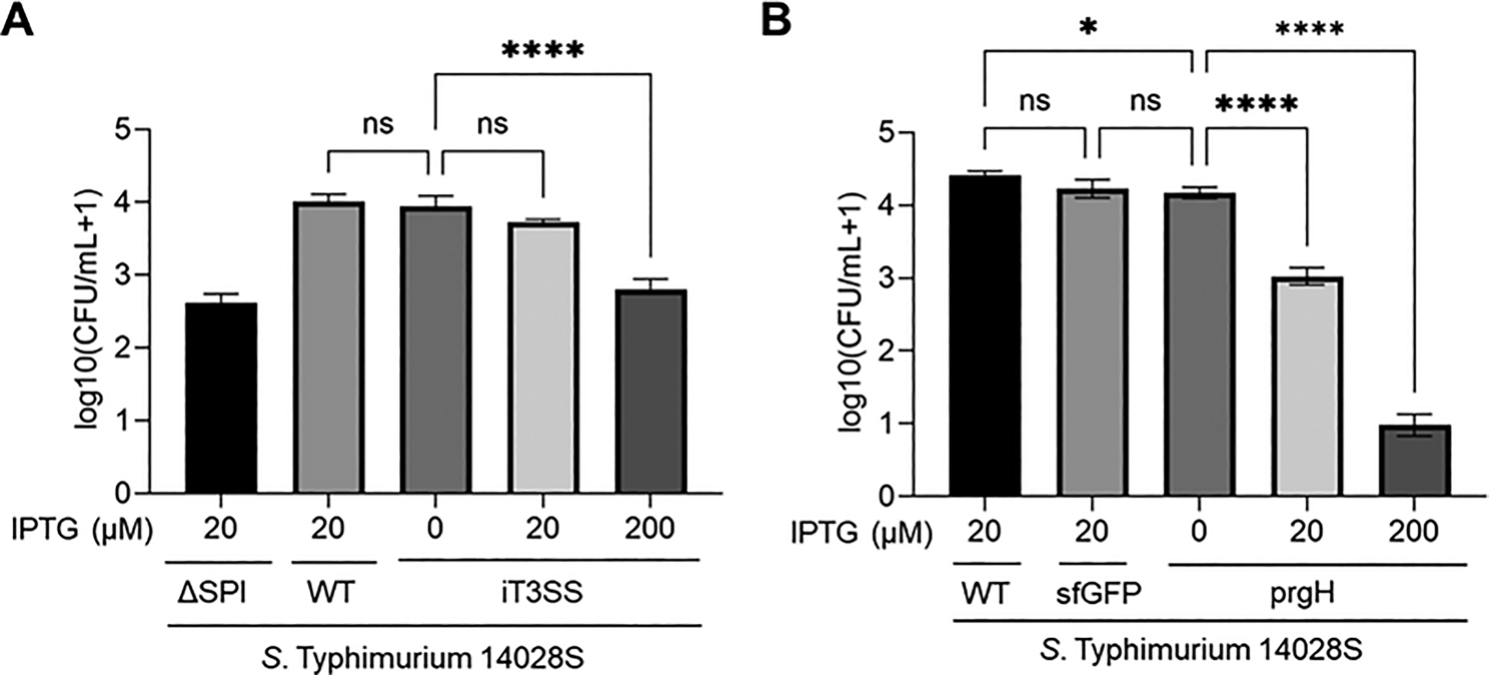
Measurement of host cell invasion efficiency by *S*. Typhimurium with suboptimal secretion machinery (**A**) *S.* Typhimurium WT strains harboring the iT3SS were cultured with varying IPTG concentrations (0, 20, and 200 µM) and assessed for invasion using the gentamicin protection assay. The ΔSPI-1 strain and the unmodified WT strain were used as negative and positive controls, respectively. (**B**) Effect of *prgH* overexpression on host cell invasion. *S.* Typhimurium 14028s strains carrying pSEVA234::prgH were grown to the exponential phase in LB supplemented with 0, 20, or 200 µM IPTG, and subsequently tested for invasion efficiency. As controls, the WT strain and sfGFP-expressing strain were cultured, respectively, under the same conditions with 20 µM IPTG and included in the assay. CFU values are presented as log10(CFU/mL + 1). Error bars represent mean ± SD (*n* = 3). Statistical analysis was performed using one-way ANOVA with Dunnett’s multiple comparison test. *P < 0.05, ****P < 0.0001, ns: not significant (*P* > 0.05)

**Fig. 5:**
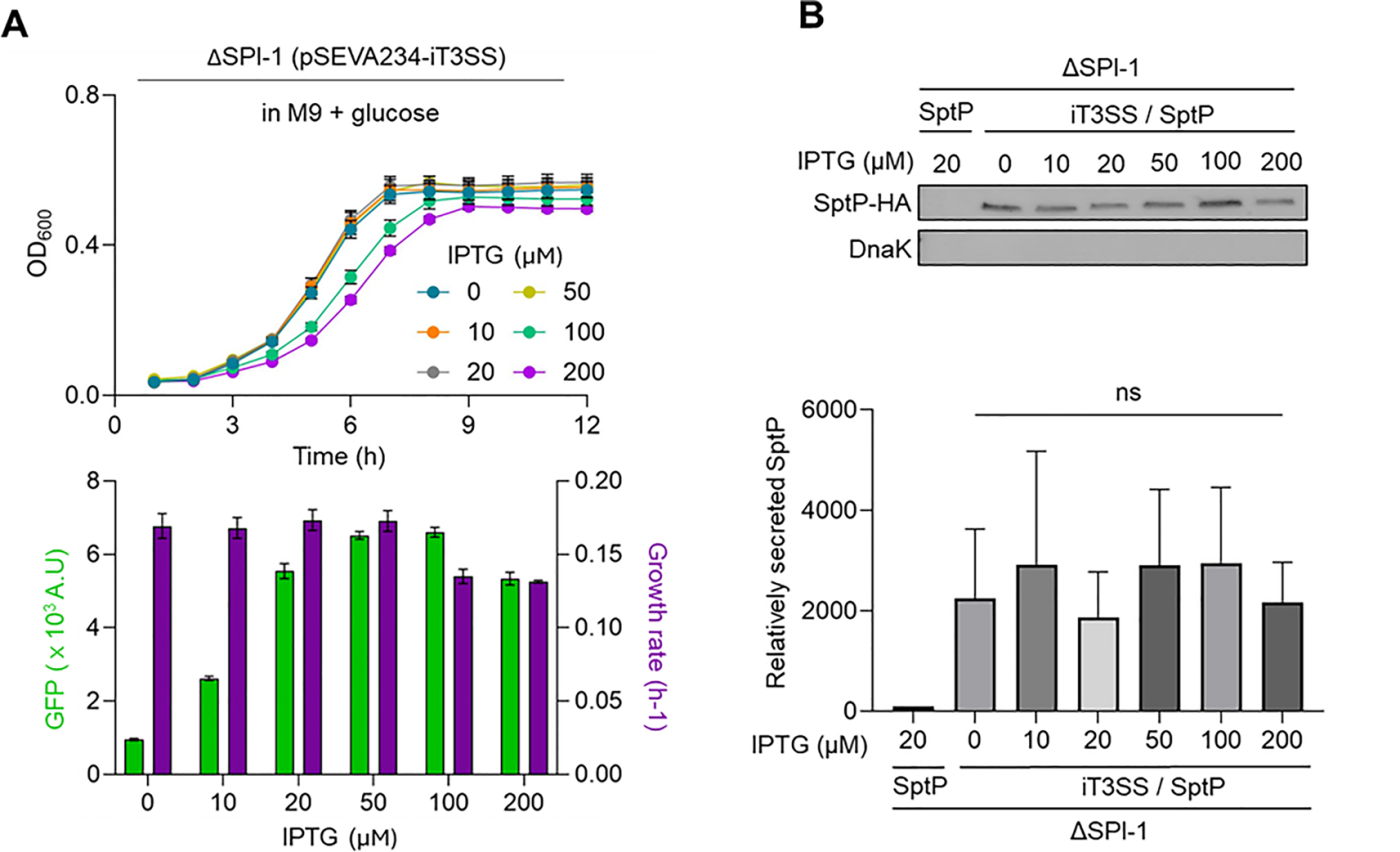
Assessment of iT3SS activity in nutrient-limited media (**A**) Monitoring of growth and fluorescent activity in the ΔSPI-1 strain expressing iT3SS in minimal medium. The ΔSPI-1 strain harboring the iT3SS was cultured in M9 minimal medium supplemented with glucose (0.2%) and varying IPTG concentrations (0–200 µM). Both cellular growth and *prgH*-fused GFP fluorescence were monitored over time. The lower bar graph presents GFP fluorescence levels at 12 h (green) and corresponding growth rates (gray), calculated from the growth curves. (**B**) Western blot analysis of secreted SptP from the ΔSPI-1 strain co-harboring the iT3SS machinery and the SptP expression system. Cells were grown in M9 minimal medium with varying IPTG concentrations (0–200 µM). The representative blot showed SptP-HA detected in culture supernatants, with DnaK used as a cytoplasmic loading control. A strain carrying only pSEVA658::SptP-HA served as a negative control. The bar graph presented densitometric quantification of SptP-HA levels (mean ± SD). No statistically significant differences were observed between IPTG concentrations (ns: not significant, *P* > 0.05)

**Fig. 6:**
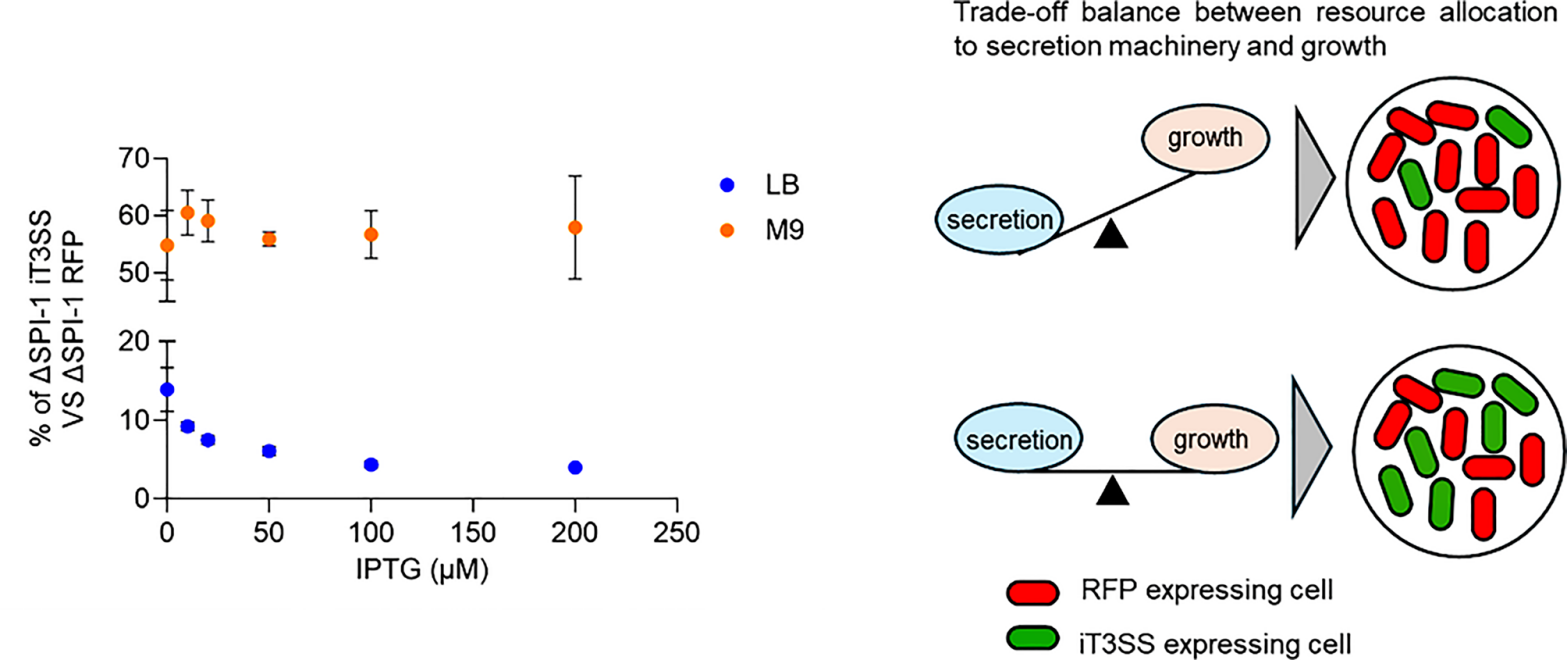
Competitive growth analysis of iT3SS-expressing strains under nutrient-rich and nutrient-limited conditions The ΔSPI-1 strain harboring the iT3SS was co-cultured with the ΔSPI-1 strain carrying a pSEVA234::RFP in either LB or M9 minimal medium supplemented with glucose (0.2%) at varying IPTG concentrations (0–200 µM) to induce the secretion system. After 24 h of co-culture, the relative abundance of each strain was quantified by flow cytometry based on RFP fluorescence signals. Data are presented as the percentage of iT3SS-expressing cells relative to the total population (mean ± SD, *n* = 3). The cartoon shows that cells that overinvest in secretion machinery show reduced competitiveness, leading to a lower proportion in mixed cultures. In contrast, cells that balance resource allocation between secretion and growth maintain competitiveness, resulting in a more stable population ratio

To examine induction dynamics, reporter strains were cultured in LB with varying IPTG concentrations. OD600 and fluorescence were recorded over 12 h (Fig. 1C). PrgH-GFP signals derived from the iT3SS increased up to 50 µM IPTG but declined at higher concentrations. In contrast, the abundance of cytoplasmic sfGFP increased linearly with IPTG concentrations (Fig. 1D). Since cell densities remained comparable across inducing conditions, this indicated that reduced PrgH–GFP levels at high induction were not due to growth inhibition. Introducing the iT3SS into the wild-type (WT) strain exhibited the same trend (Fig. S1A and S1B), demonstrating that this down regulation was independent of native SPI-1 regulation. To rule out mutation-driven loss of fluorescence, we re-cultured cells previously exposed to high IPTG in LB and observed that PrgH–GFP signals were restored when the same culture was grown under moderate IPTG conditions (Fig. S1C).

To determine whether reduced expression of the secretory apparatus affected secretion performance, a well-characterized T3SS effector, SptP [41, 42], fused with a 2×HA tag was expressed under the XylS/Pm system inducible by 3-methylbenzoate (3 MB) (Fig. 2A). Western blot analysis showed that the ΔSPI-1 strain harboring iT3SS secreted SptP-HA into the supernatant, whereas strains lacking the system did not (Fig. 2B). The cytoplasmic protein, DnaK, was absent from the supernatant, indicating that the detected SptP-HA originated from active T3SS-mediated secretion and not from cell lysis. Notably, leaky iT3SS expression without IPTG was sufficient to support detectable secretion of SptP-HA, whereas strong induction (200 µM IPTG) nearly abolished secretion and led to intracellular accumulation of SptP-HA. A similar induction-dependent suppression of secretion was observed not only in the WT strain of *S*. Typhimurium (Fig. 2C) but also in *E. coli* (Fig. S2), indicating that this phenomenon was not species-specific.

We next examined secretion of the polyester hydrolase TfCut2 [43] fused with the SptP signal peptide (Fig. S3A) to evaluate its biotechnological potential. Halo assays on polycaprolactone (PCL)-containing plates [44] revealed that cells lacking iT3SS produced only weak halos, corresponding to basal secretion, whereas moderate IPTG induction (20 µM) substantially increased halo size, indicating enhanced extracellular activity. High-level induction (200 µM IPTG) again reduced halo formation to basal levels (Fig. S3B).

Altogether, these results show that the iT3SS circuit supports controllable secretion of both native effector and heterologous proteins but exhibits induction-dependent suppression of secretion. This problem undermines the potential industrial application of the engineered system, as it results in unpredictable performance. It is therefore essential to identify the key factors underlying this limitation.

### Translational or post-translational, rather than transcriptional, burden limits the functionality of the iT3SS

We first considered that low transcription might be responsible for the low expression of iT3SS at high inducer concentration. To test this hypothesis RNA-seq analysis was performed. Since reduced secretion was observed in both WT and ΔSPI-1 strains, we conducted the analysis using the WT strain to capture the native regulatory landscape, including potential interactions with the SPI-1 network. To do so, we extracted total RNA from the WT strain carrying the iT3SS, exponentially grown in LB supplemented with 0, 20, or 200 µM IPTG, to represent basal, medium, and high induction levels, respectively. Surprisingly, the transcription of iT3SS component genes, including *prgH-GFP* fused reporter, increased proportionally with IPTG concentration (Fig. 3A). This is unlikely to result from technical artifacts or crosstalk with the host’s native T3SS genes, as the expression levels of key native regulators, such as *hilD* and *hilA* (Fig. S4A) [22, 45], as well as chromosomal T3SS structural genes, remained unchanged irrespective of IPTG treatment (Fig. S4B). Given the observed reduction of the abundance of the prgH-GFP reporter fusion despite increased transcription levels with higher inducer concentrations, we assumed that post-transcriptional regulatory mechanisms play a role in determining programmable secretion activity. In order to get a better understanding of this process a deep analysis of the functional gene categories affected by high IPTG conditions, was carried out. This analysis revealed that stress-related genes—particularly those associated with membrane stress responses—were markedly upregulated whereas ribosomal protein-coding genes were significantly downregulated, in the 200 µM IPTG-treated samples compared to the untreated control samples (Fig. 3B).

The invasion efficiency of the WT strain expressing the iT3SS was subsequently investigated using three IPTG concentrations: 0, 20, and 200 µM. We diluted overnight cultures of *S.* Typhimurium 14028s strains in fresh LB containing IPTG and incubated them for 3 h before infection. We infected HeLa cells at an MOI of 100 and assessed intracellular CFUs using a gentamicin protection assay. We included the ΔSPI-1 and WT strains as negative and positive controls for host invasion, respectively. It is noteworthy that the iT3SS construct lacked translocators (i.e., SipB, SipC, and SipD) [27]; therefore, invasion relied on the cell’s native translocators. Consequently, we determined invasion efficiency based on the capacity of the cell to synthesize the secretion machinery. The results revealed that inducing the iT3SS with 20 µM IPTG did not markedly enhance invasion efficiency compared with either the control or the non-induced samples (Fig. 4A). In contrast invasion declined by approximately one order of magnitude when 200 µM IPTG was used as inducer (Fig. 4A), mirroring the downregulation of secretory apparatus synthesis [46, 47] whereas the overproduction of GFP had no such effect (Fig. 4B).

### Strong induction of the iT3SS generates heterogeneous expression at the single-cell level

We found that the reporter activity of PrgH-GFP fusion in the iT3SS reflects the functional capacity of the programmable secretion system. Based on this observation, we further assessed the expression dynamics of the iT3SS at the single-cell level. We cultured the ΔSPI-1 strain harboring the iT3SS construct with PrgH–GFP in LB for 3 h under varying IPTG concentrations and analyzed fluorescence by flow cytometry. Surprisingly, under strong induction conditions (100 and 200 µM IPTG), the bacterial population exhibited a distinct bimodal distribution, with a subset of cells exhibiting minimal fluorescence intensity (Fig. S5). Moreover, GFP activity increased in a subset of cells in response to IPTG concentration, with greater cell-to-cell variability observed at higher induction levels (Fig. S5). This expression pattern and cellular behavior were not observed with GFP expression alone (Fig. S5). This suggests that strong activation of the iT3SS introduces expression noise, leading to bimodal or multimodal gene expression patterns at the single-cell level. Although the molecular basis of the bimodal iT3SS expression remains unclear, similar heterogeneity has been observed in native T3SS systems, where only a subset of cells activate secretion while others remain inactive [48, 49]. This division of labor can enhance population-level fitness by limiting invasion capability to a smaller subset of cells, thereby reducing overall resource consumption.

### Functionality default of the inducible iT3SS in the rich LB medium at high inducer levels does not occur in glucose minimal medium

In order to compare the expression behavior of the iT3SS genes under nutrient-limited and nutrient-rich conditions, the ΔSPI-1 strain carrying the iT3SS was cultured in M9 glucose minimal medium in the presence of various concentrations of IPTG. Both biomass yields and PrgH–GFP signals were monitored throughout the growth. While IPTG concentrations below 100 µM did not affect the overall growth yield, growth rate declined when IPTG concentrations exceeded 100 µM in glucose-amended minimal medium (Fig. 5A). In contrast to the behavior observed in cells grown in LB, the fluorescence intensities of the prgH-GFP fusion recorded at 12 h linearly increased with IPTG concentration when the cells were cultured in the glucose minimal medium. Moreover, the dramatic drop in the prgH-GFP reporter activity, noted in LB at 200 µM IPTG, did not occur under this culture condition (Fig. 5A). In parallel, secretion assays using the strain carrying the iT3SS and SptP expression plasmids, cultured in M9 minimal medium supplemented with glucose, revealed a consistent amount of SptP in the extracellular fraction across all tested IPTG concentrations (Fig. 5B). While secretion capacity was not directly regulated by the inducible system—likely due to leaky expression—the suppression observed at 200 µM IPTG in cells cultured in LB was not clearly detected in the M9 minimal medium (Fig. 5B).

Flow cytometry analysis using the reporter strain carrying the iT3SS, cultured in M9 minimal medium supplemented with glucose across an IPTG gradient, revealed that the non fluorescent sub-population (GFP-OFF) expanded as the IPTG concentration increased (Fig. S6). However, this GFP-OFF fraction was smaller, and fluorescence noise within the GFP-ON cohort was lower than that in cells grown in LB (Fig. S5).

We also performed competitive growth assays to quantify the fitness cost of iT3SS expression under different culture conditions [50, 51]. We co-cultured the iT3SS-GFP expressing strain with the WT strain expressing red fluorescent protein, either in LB or glucose minimal medium with varying IPTG concentrations to activate the secretion system. After an overnight culture, we measured the relative abundance of each strain by distinguishing its fluorescent signals using flow cytometry. In LB, the iT3SS strain’s competitiveness declined in a dose-dependent manner up to 100 µM IPTG (Fig. 6). Competitiveness remained similar at 100 and 200 µM IPTG, while PrgH–GFP fluorescence showed a clear decrease between 100 and 200 µM IPTG (Figs. 1C and 6). In contrast, no significant fitness cost was observed in minimal medium, even at high IPTG levels (Fig. 6). Although a reduction in the reporter activity was detected at 200 µM in M9 medium (Fig. 5A), the extent was far less than in LB, and with no obvious fitness costs.

## Discussion

Our study showed that the functionality of the synthetic T3SS (iT3SS) is tightly linked to the nature of the culture medium. When the bacteria grow actively in the rich LB medium high concentration of the inducer, IPTG, leads to the suppression of the secretion abilities of iT3SS, whereas this process does not occur in the glucose minimal medium M9, where the growth rate of the bacteria is low. RNA-seq analysis of LB-grown cells showed that iT3SS gene transcription increased proportionally with inducer concentration (Fig. 3A), indicating that the suppression is not attributable to insufficient transcription of the system. This suppression is thus thought to be due to translational and/or post-translational burden in the rich LB medium. This putative translational burden may be explained by the bacterial growth law of Hwa et al. that stipulates a growth rate–dependent cellular resource allocation [52, 53]. In a nutrient-rich medium, where the bacterial growth rate is high, cellular resources—especially translational capacities—are preferentially distributed to translate proteins involved in growth support. This configuration leaves little capacity for additional translational load, rendering cells less efficient to express synthetic systems [52, 53]. In contrast, under nutrient-limited conditions (e.g., minimal medium), the growth rate—and thus the resources allocated to support growth—are reduced.

Under these conditions, the cell distributes its resources more evenly between the synthesis of growth-related proteins and that of other classes of proteins. This balanced allocation supports more coordinated iT3SS translation, assembly, and membrane insertion, thereby enhancing secretion efficiency. Consistently, the fitness costs associated with iT3SS super-induction were markedly lower in glucose minimal medium than in LB.

However, not all our observations fit with this primary hypothesis. For instance, ribosomal protein abundance was downregulated in LB-grown cells under strong iT3SS induction (Fig. 3B). Ribosome and ribosomal protein biosynthesis are positively regulated by amino acid and ATP availability and conversely, reduced expression is typically associated with amino acid limitation and low energetic charge (ATP deficit) [54, 55]. Therefore, in nutrient-rich conditions, ribosomal protein expression would be expected to increase rather than decrease. The observed downregulation of ribosomal protein expression rather suggests that cells grown in LB under these conditions experience energetic stress, consistently with the high fitness costs imposed by strong iT3SS induction.

Furthermore, the strain clearly exhibits membrane stress when grown in LB medium with high inducer concentrations. Such stress may arise from improper insertion of iT3SS components into the membrane. This might be due to the fact that these components are synthesized with an imbalanced stoichiometry or that they are not folded properly. These features would compromise their assembly and thus their correct insertion into the membrane. Indeed, imbalanced expression of T3SS components is known to impair secretion-dependent functions such as host cell invasion [15, 56], highlighting the need for an accurate coordination of their expression. Misassembled or misinserted proteins are likely to be degraded by the protease machinery, which may contribute to the reduced apparent abundance of these components. Membrane stress is also associated with perturbations in membrane integrity, leading to reduced respiratory activity and diminished ATP production [57, 58]. Coping with membrane stress further consumes ATP, exacerbating the energetic deficit. Such ATP limitation could account for the decreased expression of ribosomal proteins, worsening translational saturation, and resulting in poor or unbalanced iT3SS translation. Because iT3SS assembly and secretion are highly energy-demanding processes, any reduction in ATP availability would directly impair proper components assembly and membrane insertion [59, 60]. ATP depletion may also contribute to the observed cell-to-cell variability in component abundance that would constitute an ATP-saving strategy. In addition, this energetic stress can elevate (p)ppGpp levels, shifting the cell into a stringent-response state that suppresses ribosome biogenesis and further amplifies the translational bottleneck affecting iT3SS expression [60]. These energy-related constraints, acting together with unbalanced expression of iT3SS components, are therefore likely to further limit the functionality of iT3SS in the rich LB medium.

In minimal medium, these processes would not occur since the transcription of the iT3SS components is likely to be lower. The saturation of the translational apparatus would not occur, allowing a more regular and balanced (stoichiometric) translation of the iT3SS components, promoting their smooth folding and assembly and thus their good insertion into the membrane. Indeed, fitness costs, likely linked to stress and ATP deficit, were shown to be much lower in minimal medium than in LB medium.

A comprehensive understanding of these processes including resource allocation across culture conditions will require systematic transcriptomic and proteomic comparisons between LB and minimal medium. These analyses will also reveal whether other underlying constraints play a role in the system’s performance. We consider to investigate these aspects in a follow-up study.

## Conclusions

In this study, we show that the performance of a synthetic T3SS (iT3SS) is strongly influenced by the nutritional environment in which the engineered bacteria are grown. In rich LB medium, high-level induction of the iT3SS compromises its functionality. This dysfunction is likely caused by saturation of the translational machinery, leading to reduced and/or imbalanced production of essential structural components and ultimately preventing their proper assembly and membrane insertion. In contrast, under nutrient-limited conditions, such saturation does not occur, allowing the system to remain functional even under strong induction. In this condition the fitness burden associated with the overexpression of the secretion machinery does not exist. Our study thus demonstrated that iT3SS secretion activity can be modulated simply by altering medium composition. Therefore, medium composition should be considered a key parameter when engineering secretion systems for diverse biotechnological applications. Altogether, these findings suggest that appropriate expression tuning is essential for maximizing the efficiency and reliability of programmable bacterial protein secretion platforms. Medium composition-aware design strategy for secretion system optimization can be broadly applied for the advancement of protein export technologies in synthetic biology and biotechnology.

## Electronic Supplementary Material

Below is the link to the electronic supplementary material.

Supplementary Material 1

## Data Availability

The datasets used and analyzed during the current study are available from the corresponding author on reasonable request.

## Abbreviations

T3SS: Type III Secretion System
IPTG: Isopropyl β-D-1-thiogalactopyranoside
LB: Luria-Bertani broth
Gm: Gentamycin
Km: Kanamycin
3MB: 3-Methylbenzoate
msfGFP: monomeric superfolder Green Fluorescent Protein
TCA: Trichloroacetic Acid
PVDF: Polyvinylidene Difluoride
TBST: Tris-Buffered Saline with Tween-20
ANOVA: Analysis of Variance
DMEM: Dulbecco’s Modified Eagle Medium
FBS: Fetal Bovine Serum
WT: Wild Type
MOI: Multiplicity of Infection
DPBS: Dulbecco’s Phosphate-Buffered Saline
CFUs: Colony Forming Units
RFP: Red Fluorescent Protein
GFP: Green Fluorescent Protein
