# Supplementary material for: Medium dependent factors govern the functionality of engineered type III secretion systems
Source: J Biol Eng. 2025 Dec 16;20:9. doi: 10.1186/s13036-025-00600-1 (PMC12822097; doi:10.1186/s13036-025-00600-1)

**Supplementary Table S1. Bacterial strains and plasmids used in this study**

| Strain | Description | | Reference |
| --- | --- | --- | --- |
| *E. coli* TOP10 | Strain for cloning; F–mcrA Δ(mrr–hsdRMS–mcrBC) φ80lacZΔM15 ΔlacX74 recA1 araD139 Δ(ara–leu)7697 galU galK λ–rpsL(StrR) endA1 nupG | | Invitrogen |
| *E. coli* MG1655 | Strain for gene expression analysis; wild-type K-12 derivative | | Lab stock |
| *Salmonella* Typhimurium 14028s | Wild-type strain used for T3SS expression and secretion assays | | Lab stock |
| *Salmonella* Typhimurium 14028s ΔSPI1 | SPI-1 operon deletion mutant; used for heterologous iT3SS expression | | This work |
| Plasmid | | Description | Reference |
| pSEVA234 | Cloning vector; *oriV* (pBBR1); standard multiple cloning site; Km^R^ | | (63) |
| pSEVA234::sfGFP | pSEVA234 derivative; an *sfGFP* gene cloned into the *Bam*HI/*Hind*III restriction enzyme site | | (64) |
| pSEVA234::RFP | pSEVA234 derivative; an *RFP* gene cloned into the *Avr*II/*Xba*I restriction enzyme site | | (65) |
| pSEVA234::iT3SS | pSEVA234 derivative; an iT3SS genetic circuit cloned using the isothermal assembly method | | This work |
| pSEVA234::prgH | pSEVA234 derivative; a *prgH* gene cloned into the *Bam*HI/*Hind*III restriction enzyme site | | This work |
| pSEVA658 | Cloning vector; *oriV* (RSF1010); standard multiple cloning site; Gm^R^ | | Lab stock |
| pSEVA658::SptP–2HA | pSEVA658 derivative; an *SptP* gene with *SicP* and 2× HA cloned into the *Bam*HI/*Hind*III restriction enzyme site | | This work |
| pSEVA658::SicP–TfCut2 | pSEVA658 derivative; an *SptP* gene with *SicP* and 2× HA cloned into the *Bam*HI/*Spe*I restriction enzyme site | | This work |

**Supplementary Table S2. Primers used in this study**

| Primer | Sequence (5′→3′) |
| --- | --- |
| 1-F | ATTTCACACCCTAGGCCGCG |
| 1-R | GCAGAACCGCAATGACCTCA |
| 2-F | TGAGGTCATTGCGGTTCTGC |
| 2-R | TCTTTTCATCGCCACCCGTC |
| 3-F | GACGGGTGGCGATGAAAAGA |
| 3-R | AGATTGATCACATCTTTCTC |
| 4-F | GAGAAAGATGTGATCAATCT |
| 4-R | GGGATCTTCTCGGAGCTATA |
| 5-F | TATAGCTCCGAGAAGATCCC |
| 5-R | ACATGCCCTGCCGAGTCGGA |
| 6-F | TCCGACTCGGCAGGGCATGT |
| 6-R | GTTTTCCCAGTCACGACGCG |
| prgH-F | CGCGGATCCTACTAGAGAAAGAGGAGAAATACTAGATGGAAACATCAAAAGAGAAGAC |
| prgH-R | CGCAAGCTTTTAAAGTGGGCTTGGGAAAT |
| SptP-F | CGCGGATCCTACTAGAGAAAGAGGAGAAATACTAGATGCAAGCACACCAGGATATTATCG |
| SptP-R | CGCAAGCTTTCAAGCGTAGTCAGGTACGTCGTAAGGGTAAGCGTAATCCGGAACGTCGTACGGATAGCTTGCCGTCGTCATAAGCAACTGG |
| SicP-F | GCGCGAATTCGAGCTCGGTACCCGGGGATCCTACTAGAGAAAGAGGAGAA |
| SicP-R | CCACGTTCGTAGGGGTTGGCACTTTCTGCTCCAACATCGT |
| TfCut2-F | ACGATGTTGGAGCAGAAAGTGCCAACCCCTACGAACGTGG |
| TfCut2-R | TTACTGGATCTATCAACAGGAGTCCAAGACTAGTTTAGAATGGGCAGGTC |
| Del-SpI-1 hilD-F | TACTGAAACAGTAGATTCTATCCTAACGACTTGTATTAGTTGTAGGCTGGAGCTGCTTCG |
| Del-avrA to invH-R | TAATTATATCATGATGAGTTCAGCCAACGGTGATATGGCCCATATGAATATCCTCCTTAG |

**Figure S1. Suppression of the iT3SS expression in the WT strain of *S.* Typhimurium** (A) Growth profiles of *S.* Typhimurium 14028s carrying pSEVA234::iT3SS cultured in LB with varying IPTG concentrations (0–200 μM). OD600 was measured every hour for 12 h using a microplate reader. (B) GFP fluorescence intensity at 12 h (Green bars) and corresponding growth rates (Purple bars) under each IPTG concentration. (C) Fluorescence intensity of cells pre-cultured in 200 µM IPTG and subsequently re-inoculated into fresh medium containing the indicated IPTG concentrations to rule out the possibility of spontaneous mutations under high metabolic burden conditions. Error bars represent mean ± SD (n = 3).

**Figure S2. Induction-dependent suppression of the iT3SS-mediated effector secretion in *E. coli*** Western blot analysis of SptP-HA in extracellular (supernatant) and intracellular (cell lysate) fractions of *E. coli* MG1655 strains carrying either pSEVA234::iT3SS/ pSEVA658::SptP-HA or pSEVA658::SptP-HA alone. Cells were cultured in LB with varying IPTG concentrations (0, 10, 20, and 200 μM) for iT3SS induction, followed by 3-methylbenzoate (3MB; 1 mM) induction for SptP expression. DnaK was used as a cytoplasmic loading control. The western blot shown is representative of results obtained from three independent experiments.

**Figure S3. Excessive induction of iT3SS impairs the secretion efficiency of the heterologous plastic-degrading enzyme** (A) Schematic representation of the cutinase (TfCut2) expression system for the T3SS-dependent secretion. The TfCut2 coding gene was fused to the SptP-derived T3SS signal sequence and cloned into the pSEVA658 plasmid. The resulting plasmid was introduced into either the ΔSPI-1 strain carrying the iT3SS or the ΔSPI-1 strain lacking the system. (B) Polycaprolactone (PCL) degradation assay evaluating the secretion of TfCut2. The ΔSPI-1 strain expressing TfCut2, with or without the iT3SS, was cultured in the presence or absence of IPTG, and the filtered supernatants were spotted onto PCL agar plates. Clear halos indicate enzymatic PCL degradation. Halos produced by supernatants from strains lacking iT3SS represent the basal level of TfCut2 activity, likely resulting from unintended secretion.

**Figure S4. Effect of iT3SS induction on chromosomal T3SS gene expression.** RNA-seq of *S.* Typhimurium 14028s (pSEVA234::iT3SS) grown in LB with 0, 20, or 200 μM IPTG. Expression of (A) *hilD* and *hilA* and (B) SPI-1 structural genes is shown as bar graphs or violin plots, respectively, based on log₂-transformed RLE values. Error bars reflect biological variation (n = 3). Statistical significance was determined using an unpaired Student’s *t*-test and one-way ANOVA with Dunnett’s multiple comparison test (ns: not significant, P > 0.05).

**Figure S5. Effect of iT3SS induction in LB at the single-cell level.** The ΔSPI-1 strain carrying either pSEVA234::iT3SS or pSEVA234::sfGFP was grown overnight in LB, diluted 1:20 into fresh LB containing various IPTG concentrations (0, 10, 20, 50, 100, and 200 μM), and incubated for 3 h before analysis. A non-GFP control strain lacking both the iT3SS and sfGFP expression systems was also included. Fluorescent activity was analyzed by flowcytometry. The GFP-off population was defined by FITC values lower than the threshold marked by the dotted line. The histograms shown are representative of three independent experiments.

**Figure S6. Effect of iT3SS induction in minimal medium at the single-cell level.** Reporter strains were grown in M9 minimal medium supplemented with glucose, following the same procedure described in Figure S5. Fluorescent protein expression was measured by flow cytometry. The GFP-off population was defined as cells with FITC fluorescence below the threshold indicated by the dotted line. The histograms shown are representative of three independent experiments.

**Figure S1**


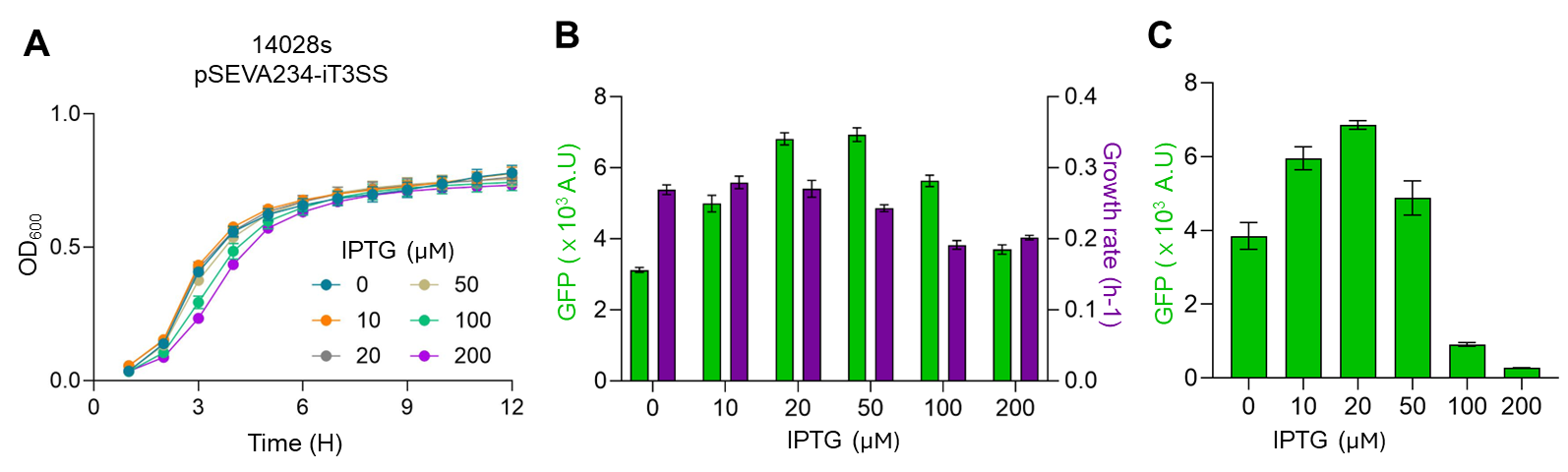


**Figure S2**


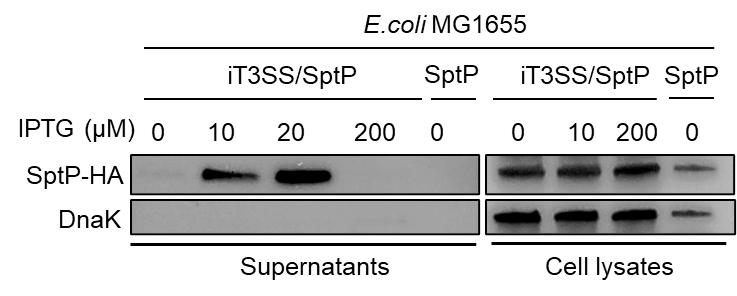


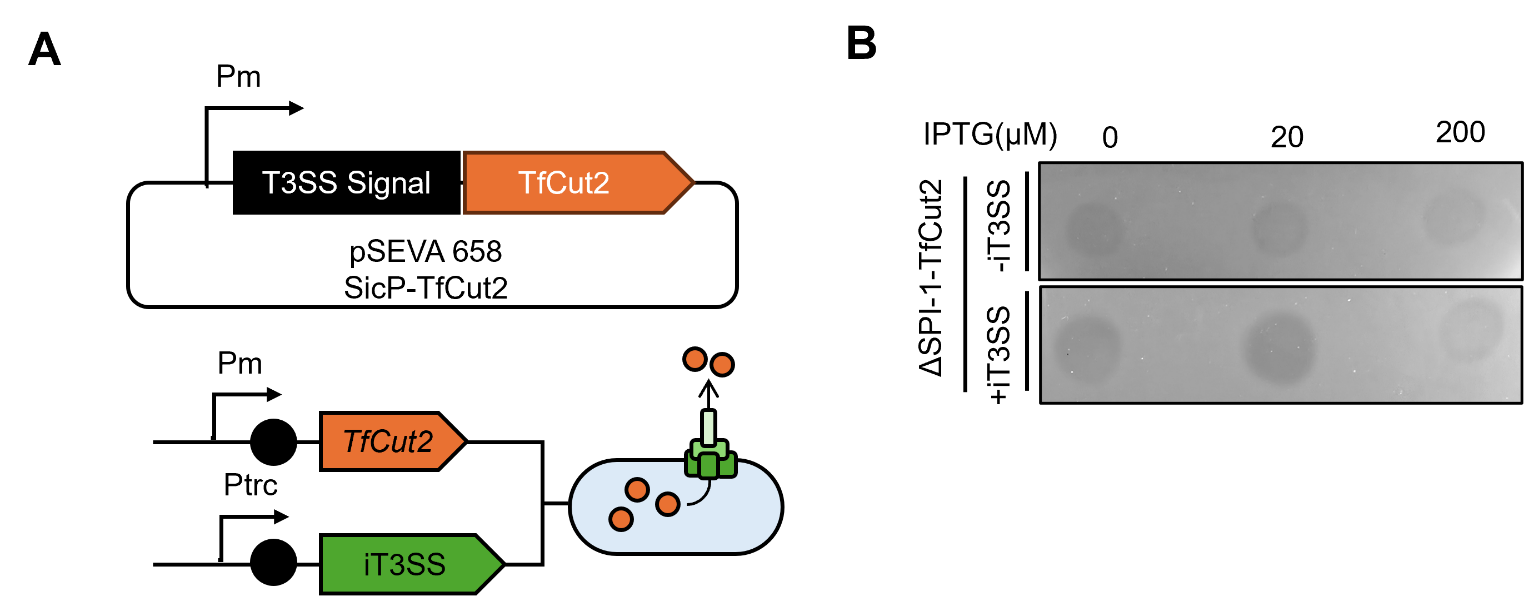
**Figure S3**


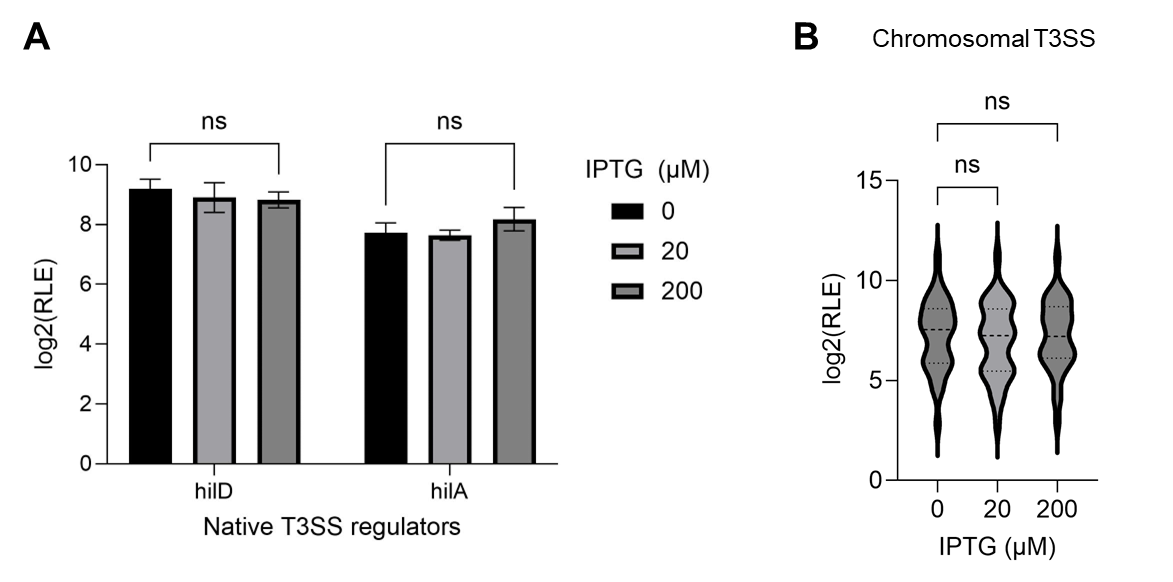
**Figure S4**

**Figure S5**


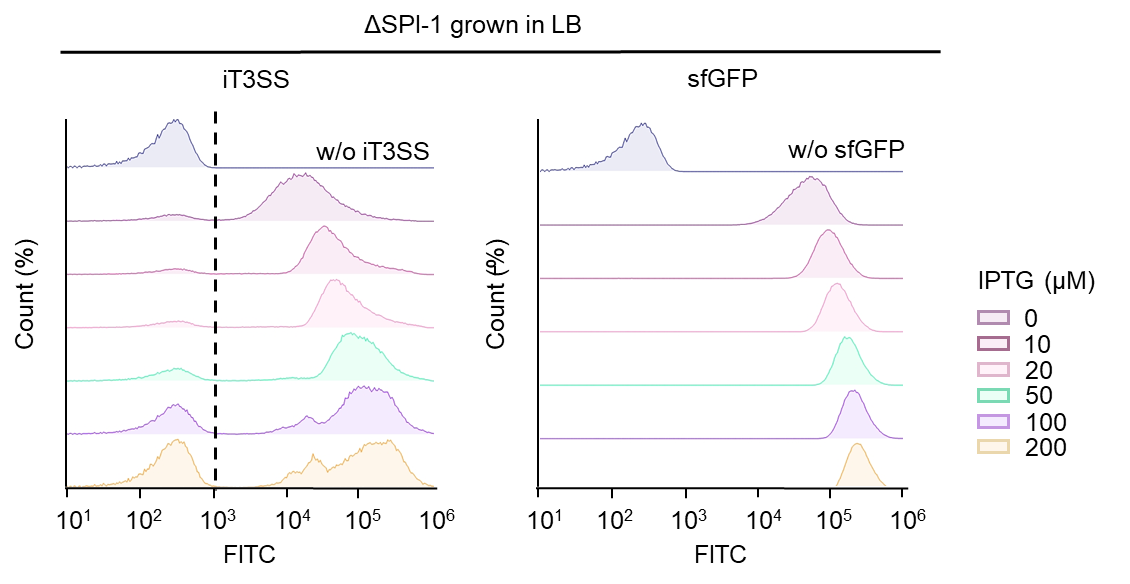


**Figure S6**


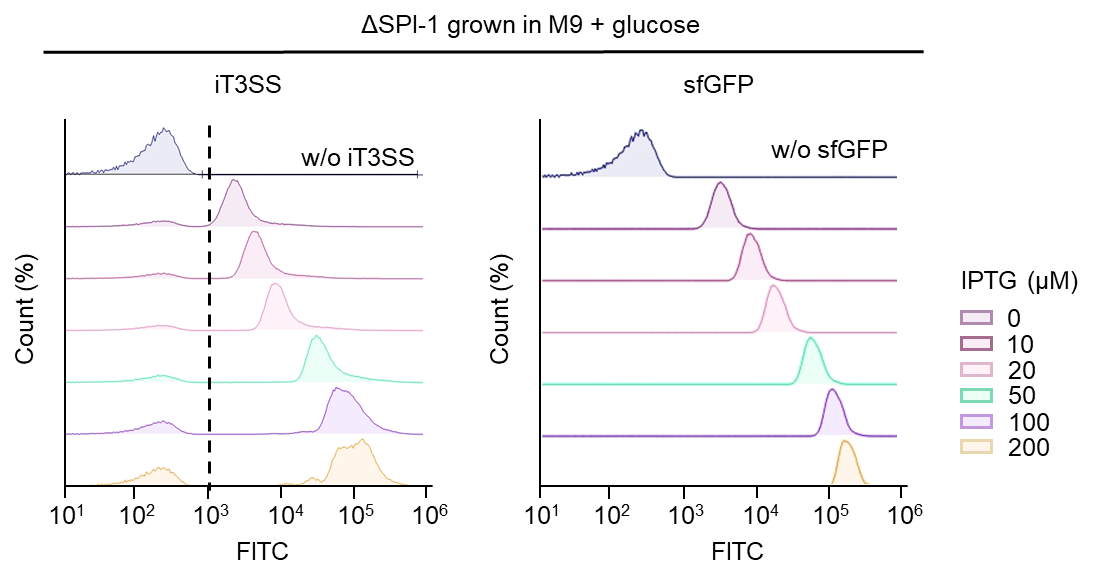

Supplement: Supplementary file 1 — Supplementary Material 1 [file 13036_2025_600_MOESM1_ESM.docx]
